# Diallyl trisulfide attenuates hyperglycemia-induced endothelial apoptosis by inhibition of Drp1-mediated mitochondrial fission

**DOI:** 10.1007/s00592-019-01366-x

**Published:** 2019-05-21

**Authors:** Ying Hao, Hui-Min Liu, Xin Wei, Xue Gong, Zhao-Yang Lu, Zhen-Hao Huang

**Affiliations:** 1 Department of Cardiology, Shanghai East Hospital, Tongji University, 1800 Yuntai Road, Shanghai, 200126 China; 2 Department of Hematology, The Second Hospital of Shanxi Medical University, 382 Wuyi Road, Taiyuan, 030001 China; 3 Department of Pharmacy, Xinhua Hospital, Shanghai Jiaotong University School of Medicine, 1665 Kongjiang Road, Shanghai, 200092 China; 4 Department of Cardiology, Delta Health Hospital, 109 Xule Road, Shanghai, 201702 China; 5 Department of Cardiology, The Second Hospital of Shanxi Medical University, 382 Wuyi Road, Taiyuan, 030001 China

**Keywords:** Diallyl trisulfide, High glucose/hyperglycemia, Endothelial cells, Apoptosis, Mitochondrial fission, Drp1

## Abstract

**Aims:**

Hyperglycemia induces endothelial cell apoptosis and blood vessel damage, while diallyl trisulfide (DATS) has shown cardiovascular protection in animal models and humans. The aim of this study was to investigate the effects of DATS on inhibition of high glucose-induced endothelial cell apoptosis and the underlying molecular events.

**Methods:**

Human umbilical vein endothelial cells (HUVECs) were incubated with DATS (100 μM) for 30 min and then cultured in high-glucose medium (HG, 33 mM) for 24 h for assessment of apoptosis, glutathione (GSH), reactive oxygen species (ROS), superoxide dismutase (SOD), and gene expression using the terminal deoxyuridine triphosphate nick end labeling (TUNEL), flow cytometry, caspase-3 activity, ROS, SOD, and western blot assays as well as JC-1 and MitoTracker Red staining, respectively.

**Results:**

DATS treatment significantly inhibited high glucose-induced HUVEC apoptosis by blockage of intracellular and mitochondrial ROS generation, maintenance of the mitochondrial membrane potential, and suppression of high glucose-induced dynamin-related protein 1 (Drp1) expression. Furthermore, DATS blockage of high glucose-induced mitochondrial fission and apoptosis was through adenosine monophosphate-activated protein kinase (AMPK) activation-inhibited Drp1 expression in HUVECs.

**Conclusions:**

DATS demonstrated the ability to inhibit high glucose-induced HUVEC apoptosis via suppression of Drp1-mediated mitochondrial fission in an AMPK-dependent fashion.

## Introduction

Diabetes mellitus consists of a group of metabolic disorders with an increased blood sugar level (namely hyperglycemia) due to either insufficient insulin production by the pancreas (type I diabetes) and/or cells not responding to insulin (insulin-resistant type II diabetes) in the body [1]. Diabetes increases the risk of long-term complications, one of which is damage to the blood vessels, which doubles the risk of cardiovascular disease development [2]. In addition, approximately 75% of deaths in diabetics are due to coronary artery disease [3]. Diabetes patients also have shown an increased incidence of atherosclerosis [4]. Endothelial dysfunction is considered as a pivotal step in atherosclerosis occurrence in diabetes patients [5]. Previous studies have shown that hyperglycemia in diabetes patients leads to endothelial dysfunction and increased production of reactive oxygen species (ROS) [6, 7]. Nicotinamide adenine dinucleotide phosphate oxidases, uncoupled nitric oxide synthases (eNOS), and the mitochondria are the main sources of ROS production [8]. Indeed, previous studies have demonstrated that superabundant generation of mitochondrial ROS plays a key role in initiation and development of endothelial dysfunction [9, 10]. The mitochondria are the key modulator of energy generation, ROS production, signal transmission, and apoptosis mediation in cells, while the mitochondrial energetic state is closely related to the mitochondrial morphology [11]. For example, mitochondrial fission and fusion are essential in the maintenance of their organelle fidelity, and excessive mitochondrial fission has been revealed to be detrimental and to contribute to cell apoptosis because they lead to superabundant fragmented mitochondria and mitochondrial ROS generation in mammalian cells [12]. Furthermore, hyperglycemia has been shown to induce endothelial cell apoptosis [13]. Thus, further research on hyperglycemia-induced endothelial cell damage could lead to the discovery of novel strategies to protect endothelial cells and to reduce diabetes-stimulated long-term complications.

Garlic consumption is inversely associated with the progression of cardiovascular disease in patients [14]. Diallyl trisulfide (DATS) is a garlic-derived organosulfur compound that possesses a variety of well-documented pharmacological activities, including cardiovascular protective effects through inhibition of hyperglycemia-induced vascular endothelial injury, attenuation of mitochondrial oxidative stress, and prevention of hyperglycemia-induced cardiac apoptosis; at the gene level, DATS has been shown to activate the insulin-like growth factor 1 receptor/p-protein kinase B (Akt) signaling pathway and to regulate the expression of ROS-generating enzymes [15, 16]. Our recent study also has revealed that DATS was able to induce tissue angiogenesis in a diabetic mouse model of hind limb ischemia [17]; such an effect was independent of the antihyperglycemic activity of DATS, since other hypoglycemic agents, including insulin, showed fewer cardiovascular protective effects [17]. In addition, a recent study has shown that the effects of DATS prevention of myocardial ischemia–reperfusion (MI/R) injury-induced cardiomyocyte apoptosis were through adenosine monophosphate-activated protein kinase (AMPK) activation in streptozotocin-induced diabetic rats [18]. Indeed, AMPK activation has demonstrated a pivotal role in suppression of MI/R injury-induced cardiomyocyte apoptosis [19]. However, it remains unknown whether mitochondrial fission is involved in the antiendothelial cell apoptosis effect of DATS in the hyperglycemic state. Therefore, the aim of the present study was to investigate whether DATS can alleviate endothelial cell apoptosis induced by hyperglycemia via inhibition of mitochondrial fission as well as the underlying molecular mechanism(s). The results of this study are expected to provide insightful information for the future use of DATS as a protective agent in the control of diabetes-induced cardiovascular complications.

## Materials and methods

### Cell lines, culture, and treatment

The human umbilical vein endothelial cells (HUVECs) used in this study were originally from the American Type Culture Collection (Cat. CRL1730; Manassas, VA, USA) and cultured in Dulbecco’s modified Eagle medium (DMEM) supplemented with 10% fetal bovine serum, 100 U/mL penicillin, and 0.1 mg/mL streptomycin in a humidified incubator with 5% CO_2_ at 37 °C. In our experiments, HUVECs at passage 3–4 were treated as follows: (1) normal 5 mM glucose (NG); (2) high 33 mM glucose (HG); (3) HG plus DATS (100 μM, based on our previous investigation); (4) HG plus Mito-TEMPO (2 μM; a mitochondria-targeted antioxidant); (5) HG plus si-dynamin-related protein 1 (Drp1); (6) HG plus DATS and Drp1; (7) HG plus (Ad)-AMPK-CA; and (8) HG plus DATS and siAMPK. In these experiments, HUVECs were pretreated with DATS or Mito-TEMPO for 30 min before HG was added, and the cells were transfected with different siRNAs or plasmids for 48 h to knock down or overexpress target genes, respectively. After that, HUVECs were harvested to determine the corresponding test index, while HG culture was used to mimic the hyperglycemic microenvironment according to a previous study [13].

### Terminal deoxynucleotidyl transferase dUTP-biotin nick end-labeling (TUNEL) assay

After treatment of HUVECs on coverslips for 24 h, the level of apoptosis in HUVECs was determined by a TUNEL assay using the In situ Cell Death Detection Kit (Roche Molecular Biochemicals, Mannheim, Germany), according to the manufacturer’s protocol. The percentage of apoptotic endothelial cells was quantified by using a fluorescence microscope (Olympus, Tokyo, Japan).

### Flow cytometry analysis

After the HUVECs were treated for 24 h, they were detached with 0.25% trypsin and washed three times with cold phosphate-buffered saline. HUVECs were resuspended in 500 µL of binding buffer and stained with 5 µL of Annexin V plus 5 µL of propidium iodide (PI; Annexin V/PI Apoptosis Detection kit; BD Biosciences) for 15 min at room temperature in the dark. Apoptosis was determined with a FACScan flow cytometer (BD Biosciences), and the data were analyzed by Modfit 3.0.

### Caspase-3 activity assay

After treatment for 24 h, the HUVECs were lysed using radioimmunoprecipitation assay buffer (RIPA buffer; Beyotime, Shanghai, China) and quantified using the bicinchoninic acid (BCA) protein assay kit (Beyotime), according to our previous study [20]. Caspase-3 activity was assessed by a Caspase-3 activity assay kit (Beyotime), according to the manufacturer’s instructions.

### Measurement of ROS production

After treatment for 24 h, the intracellular ROS level was measured using a Reactive Oxygen Species Assay Kit (Abcam, Cambridge, UK), according to the kit’s instructions. The images were captured by a fluorescence microscope (Olympus) at an excitation of 484 nm and an emission of 530 nm. In addition, the MitoSOX Red mitochondrial superoxide indicator (Invitrogen, Carlsbad, CA, USA) was employed to measure mitochondria in live cells, as recommended by the manufacturer. The images were obtained under a fluorescence microscope (Olympus) at an excitation of 488 nm and an emission of 583 nm. The fluorescence intensity was analyzed by ImageJ software (National Institutes of Health, Bethesda, MD, USA) in at least five fields and normalized by the number of cells.

### Measurement of the superoxide dismutase (SOD) activity and glutathione (GSH) level

After treatment for 24 h, the total cellular protein of each group was lysed using RIPA buffer and quantified using a BCA protein assay kit (Beyotime) for detection of the SOD and GSH levels in the supernatant using a SOD kit (Beyotime) and a GSH assay kit (Beyotime), respectively. The data were obtained using a SpectraMax Plus384 spectrophotometer (Molecular Device, Sunnyvale, CA, USA), according to the manufacturer’s instructions.

### Measurement of the mitochondrial membrane potential

After treatment for 24 h, the mitochondrial membrane potential in HUVECs was measured by using the JC-1 reagent (Beyotime), an ideal mitochondrial membrane potential detection fluorescent probe, according to the manufacturer’s instructions. The images and fluorescence levels were acquired by using a fluorescence microscope (Olympus) at an excitation of 488 nm and the registration of both green and red fluorescence. The fluorescence intensity was analyzed by ImageJ software.

### Extraction of mitochondrial and cytoplasmic protein

After treatment for 24 h, each group of cells was collected and mixed with 1 mL of mitochondrial separation reagent (Sangon Biotech Co., Shanghai) to gently suspend the cells. The cells were placed on ice for 15 min, and the suspension was homogenized 30 times. Then, the cell homogenate was centrifuged at 10,000 rpm for 10 min (4 °C), the supernatant was collected and centrifuged at 11,000 rpm for 10 min (4 °C), and finally, the supernatant was collected and centrifuged at 12,000 rpm for 10 min (4 °C). Cytoplasmic proteins without mitochondria were obtained. Approximately 100 μL of the mitochondrial solution was added to the mitochondrial pellet, the mixture was placed on ice for 30 min, then the mixture was centrifuged at 13,000 rpm for 10 min (4 °C), and the supernatant was collected to obtain the mitochondrial proteins. The cytoplasmic protein concentration was quantified using a BCA protein assay kit.

### Western blot

After treatment for 24 h, the total cellular protein of each group was lysed using RIPA buffer, the mixture was centrifuged for 10 min at 15,000 rpm (4 °C), and then, the protein concentration was quantified using a BCA protein assay kit. Equal amounts (30 μg) of protein samples were separated in sodium dodecyl sulfate–polyacrylamide gels by electrophoresis and transferred onto polyvinylidene fluoride membranes (Millipore, Billerica, MA, USA). The membranes were then subjected to western blotting using antibodies against cytochrome c (1:1000), Bax (1:1000), Bcl-2 (1:1000), Drp1 (1:1000), AMPK (1:1000), p-AMPK (1:1000), acetyl coenzyme A carboxylase (*ACC*; 1:1000), p-ACC (1:1000), voltage-dependent anion channel (1:1000), and β-actin (1:1000). The positive protein signals were detected by using the Odyssey Imaging System (LI-COR Biosciences, Lincoln, NE, USA), and the intensity of the bands was quantified by using Quantity One Software (Bio-Rad) after normalization to the β-actin control.

### Mitochondrial morphology

HUVECs were seeded onto coverslips and subjected to treatment for 24 h. After that, the cells were incubated with MitoTracker Red (25 nM, Molecular Probes) in a cell culture incubator with 5% CO_2_ at 37 °C for 30 min, and then, the fluorescence levels were captured under a confocal microscope. The mitochondrial morphology was classified into three types, i.e., tubular (> 4 μm in length), intermediate (2–4 μm in length), and fragmented (< 2 μm in length) and quantified using ImageJ software, according to previous studies [21, 22]. At least 100 cells were analyzed in each sample to determine the cells undergoing mitochondrial fragmentation.

### Construct cloning, adenovirus production, and cell infection

Adenoviruses carrying Drp1 RNA interference (AdDrp1-RNAi), AMPK cDNA (Ad-AMPK-CA), and their scrambled controls, respectively, were amplified, generating adenoviruses as previously described [23] with the technical help of Hanbio Biotech Co., Ltd. (Shanghai, China). In this study, we infected these adenoviruses into HUVECs, according to the manufacturer’s instructions and the multiplicity of infection levels. The infection efficiency was confirmed by using western blotting.

### RNA interference and Drp1 overexpression

Specific siRNA targeting AMPK (siAMPK) was purchased from Santa Cruz Biotechnology (Santa Cruz, CA, USA), while the Drp1 plasmid was purchased from Addgene (Cambridge, MA, USA). The siAMPK and Drp1 plasmids were transfected with Lipofectamine 2000 (Invitrogen, Carlsbad, CA, USA), according to the manufacturer’s instructions.

### Statistical analysis

The experiments were repeated three times, and the results were expressed as the mean ± standard error of the mean (SEM). The Kruskal–Wallis one-way analysis of variance test was employed to compare the means of the various groups, followed by Tukey’s post hoc test analysis. A value of *p* < 0.05 was considered statistically significant.

## Results

### DATS inhibition of HUVEC apoptosis under high-glucose conditions

**Fig. 1:**
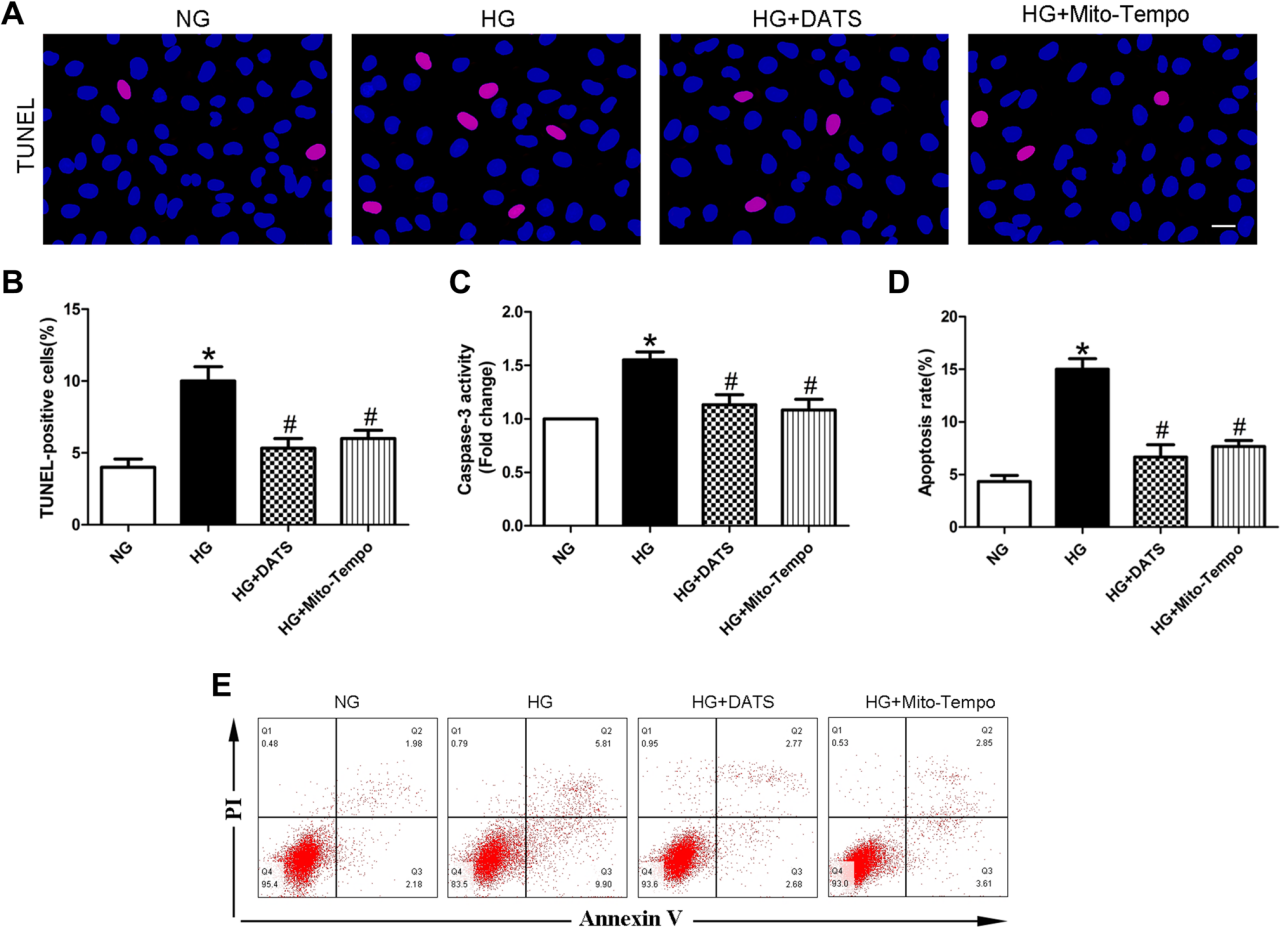
Effect of DATS on inhibition of high glucose-induced HUVEC apoptosis in vitro. **a** TUNEL assay. HUVECs were seeded, treated with different agents, then grown under normal glucose (NG) or high-glucose (HG) conditions, and subjected to the TUNEL assay. The images show DAPI-stained (blue) and TUNEL-positive cells (pink); scale bar = 25 μm. **b** Graph shows the quantified HUVEC apoptosis data. **c** Caspase-3 activity assay. HUVECs were seeded, treated with different agents, then grown under normal glucose (NG) or high-glucose (HG) conditions, and subjected to the caspase-3 activity assay. **d** Graph shows the quantified HUVEC apoptosis data determined by flow cytometry. **e** Apoptosis was determined by flow cytometry. HUVECs were seeded, treated with different agents, grown under normal glucose (NG) or high-glucose (HG) conditions, and subjected to annexin V/PI staining for flow cytometry detection. The data are expressed as mean ± SEM (*n* = 3). *p < 0.05 versus the NG group and ^#^*p* < 0.05 versus the HG group

In this study, we first assessed the effects of DATS on regulation of high glucose-induced HUVEC apoptosis. The TUNEL assay results demonstrated that HUVECs cultured under high-glucose conditions showed elevated cell apoptosis compared with that of the NG-cultured HUVECs. In contrast, the addition of DATS into the cell culture medium attenuated high glucose-induced HUVEC apoptosis. These findings were further confirmed by flow cytometry (Fig. 1a, b, d, e). In addition, the level of caspase-3 activity, an apoptotic marker, was downregulated after DATS treatment (Fig. 1c). Moreover, we performed a parallel experiment using Mito-TEMPO, a mitochondria-targeted antioxidant as a positive control, to verify the antiapoptotic effect of DATS via mitochondrial activity regulation and found that Mito-TEMPO possessed the same effect as DATS on downregulation of high glucose-induced HUVEC apoptosis (Fig. 1).

### DATS reduction in ROS generation in HUVECs under high-glucose conditions

**Fig. 2:**
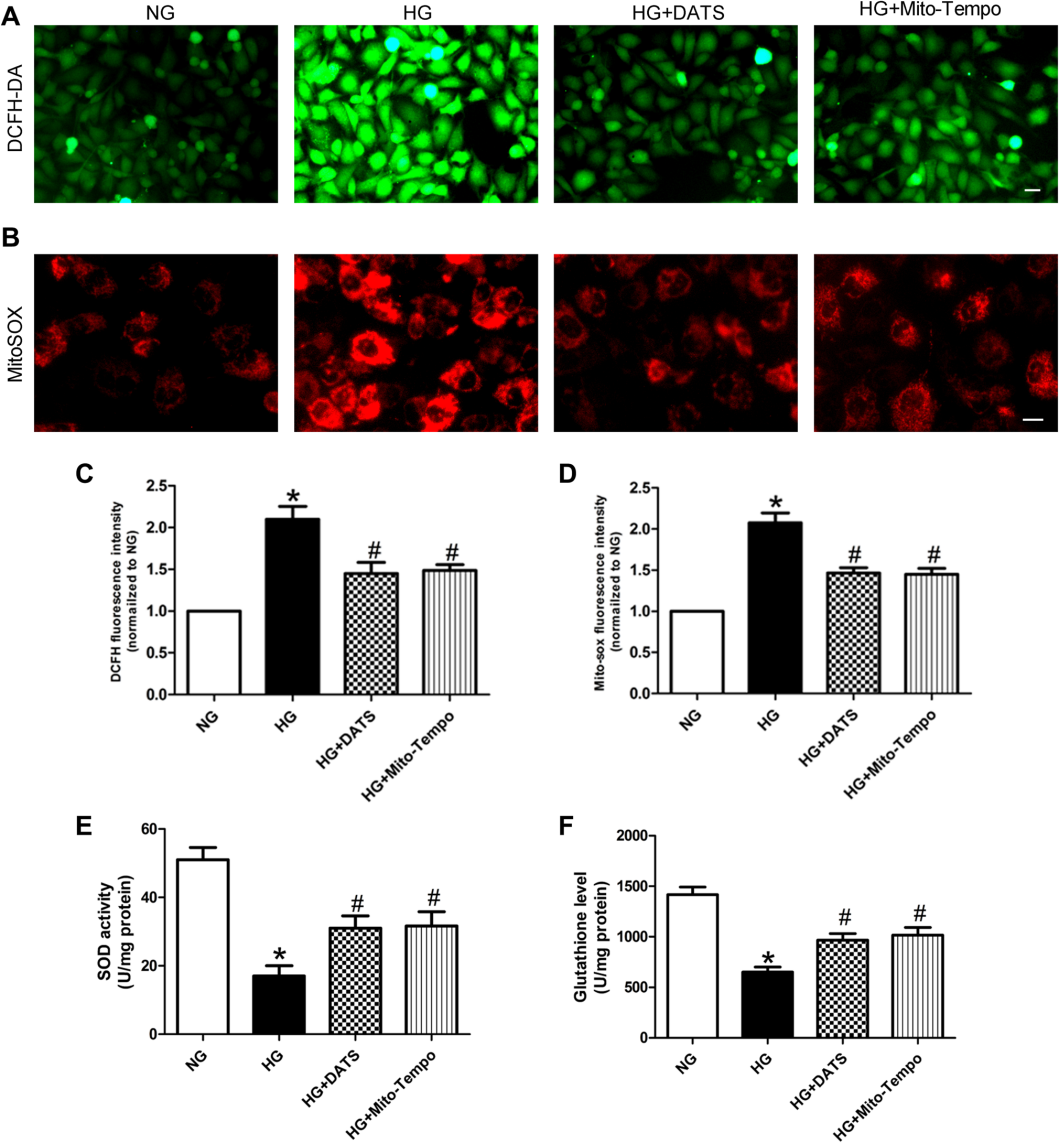
Effect of DATS on inhibition of high glucose-induced ROS generation in HUVECs. **a**, **b** Intracellular and mitochondrial ROS assay. HUVECs were seeded, treated with different agents, then grown under normal glucose (NG) or high-glucose (HG) conditions, and subjected to the assay. Representative photographs of intracellular ROS (**a**) and mitochondrial ROS (**b**). Scale bar = 25 μm. **c** Quantified data of the intracellular ROS levels. **d** Quantified data of the mitochondrial ROS levels. **e**, **f** SOD and GSH assays. HUVECs were seeded, treated with different agents, then grown under normal glucose (NG) or high-glucose (HG) conditions, and subjected to the assay. The data are represented as mean ± SEM (*n* = 3). *p < 0.05 versus the NG group and ^#^*p* < 0.05 versus the HG group

Next, we assessed the effects of DATS on regulating the ROS level in HUVECs under high-glucose culture conditions using dichlorofluorescein and MitoSOX to measure the cellular superoxide anion and mitochondrial ROS levels, respectively. Our results showed that both the cellular superoxide anion and mitochondrial ROS levels in the HG group were significantly higher than those in the NG group. In contrast, DATS and Mito-TEMPO treatment was able to lower their levels in the HG group (Fig. 2a–d). Moreover, DATS and Mito-TEMPO treatment also enhanced SOD activity and augmented the GSH level in HUVECs under high-glucose culture conditions (Fig. 2e, f).

### DATS reduction in the high glucose-induced HUVEC mitochondrial apoptotic pathway

**Fig. 3:**
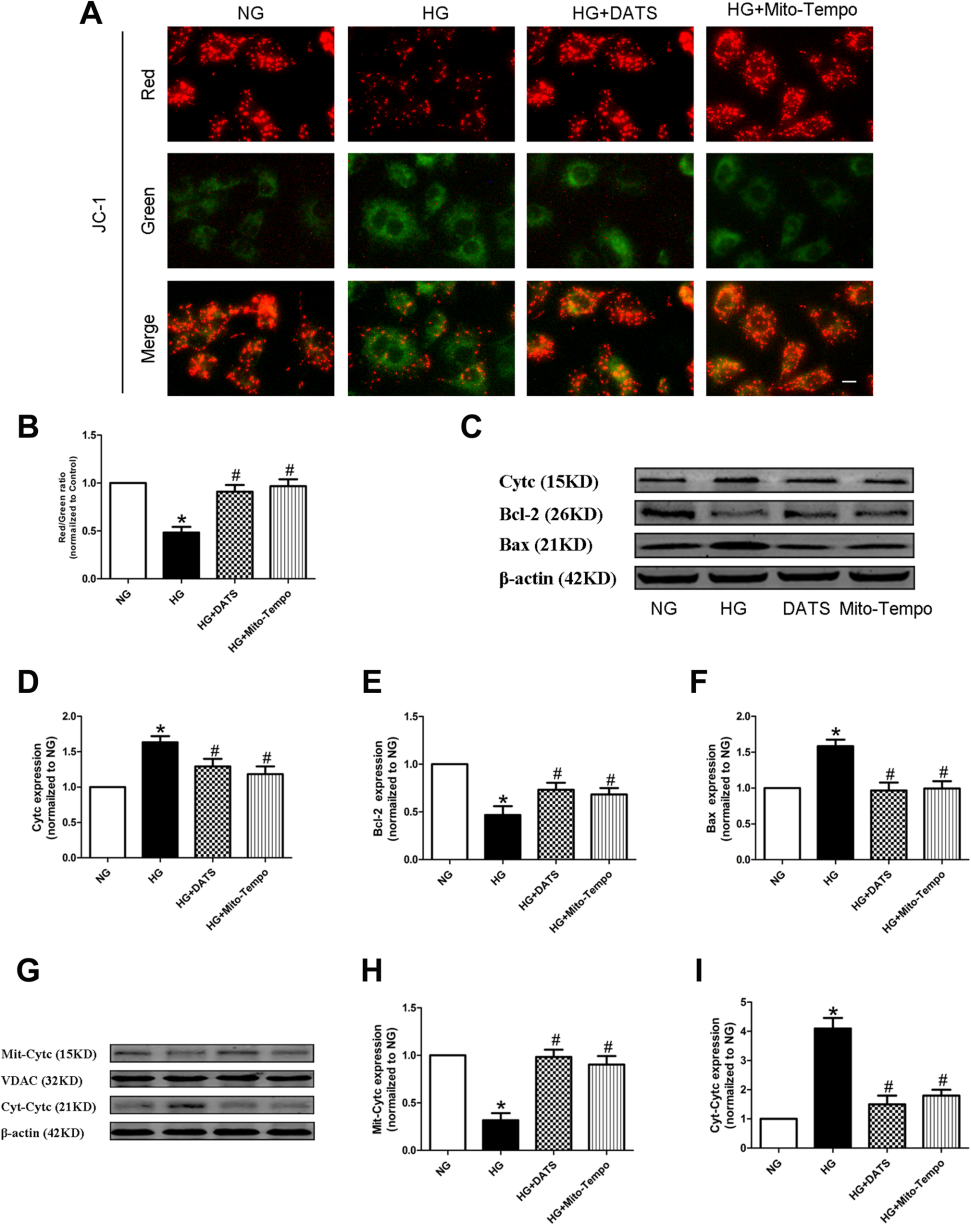
Effect of DATS on inhibition of the high glucose-induced HUVEC mitochondrial apoptotic pathway. **a** JC-1 staining of the mitochondrial membrane potential, a hallmark of mitochondrial apoptosis. HUVECs were seeded onto a coverslip, treated with different agents, then grown under normal glucose (NG) or high-glucose (HG) conditions, and subjected to the assay. Scale bar = 25 μm. **b** Quantified data described in (**a**). **c**–**f** Western blot. HUVECs were seeded, treated with different agents, then grown under normal glucose (NG) or high-glucose (HG) conditions, and subjected to western blot analysis of cytochrome c (Cytc), Bcl-2, and Bax, markers of mitochondria-related cell apoptosis. **g**–**i** Western blot. HUVECs were seeded, treated with different agents, then grown under normal glucose (NG) or high-glucose (HG) conditions, and subjected to western blot analysis of cytochrome c in the mitochondrial and cytoplasmic fractions. The data are represented as mean ± SEM (*n* = 3). *p < 0.05 versus the NG group and ^#^*p* < 0.05 versus the HG group

To assess mitochondrial injury, we detected the change in the mitochondrial membrane potential, a hallmark of such an event. We found that HG was able to significantly reduce the mitochondrial membrane potential in JC-1-stained HUVECs, compared with the NG group. In contrast, DATS and Mito-TEMPO treatment was able to robustly restore the mitochondrial membrane potential in JC-1-stained HUVECs under high-glucose culture conditions (Fig. 3a, b). The alteration of the mitochondrial membrane potential could lead to mitochondria-induced apoptosis. Thus, the levels of mitochondrial-related apoptotic markers, like cytochrome c, Bax, and Bcl-2, were also altered and restored by DATS and Mito-TEMPO in JC-1-stained HUVECs under high-glucose culture conditions (Fig. 3c, f). In addition, we also measured the levels of cytochrome c protein in mitochondrial and cytosolic fractions by western blot assays. The data showed that DATS decreased the cytosolic cytochrome c level and increased the mitochondrial cytochrome c level after HG treatment (Fig. 3g, i).

### DATS protection of the mitochondria by inhibition of Drp1-mediated mitochondrial fission

**Fig. 4:**
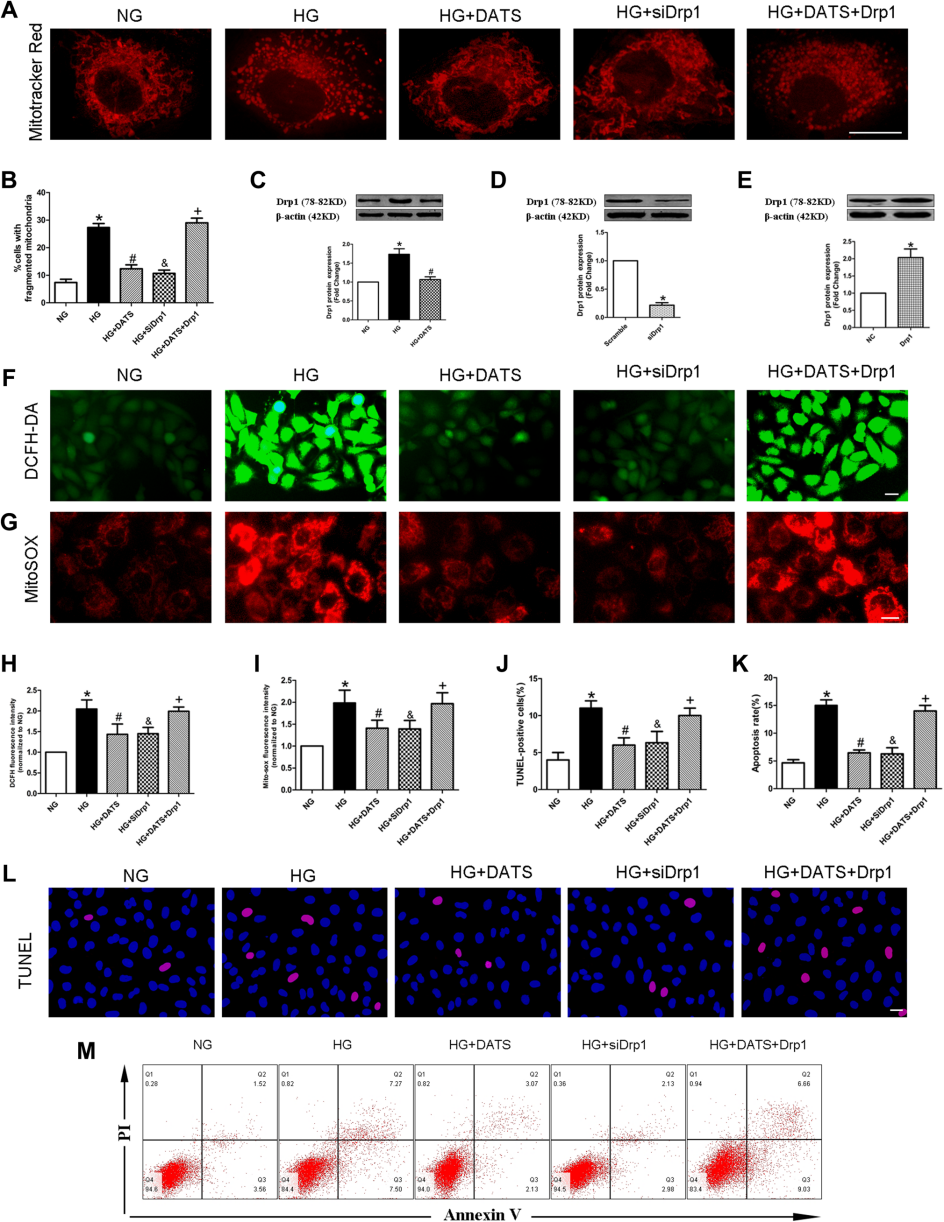
Effect of DATS on protection of high glucose-induced HUVEC mitochondrial fission via Drp1 inhibition. **a** MitoTracker red staining of the mitochondrial morphology. HUVECs were seeded, treated with different agents, and subjected to the assay. Scale bar = 25 μm. **b** Quantified data described in (**a**). **c**–**e** Western blot. HUVECs were seeded, treated with different agents, and subjected to western blot analysis of Drp1 protein. **f**–**g** The intracellular and mitochondrial ROS assay. **h**–**i** Quantified data described in (**f**) and (**g**). **i**–**k** Quantified data described in (**l**) and (**m**). **l**–**m** TUNEL assay and flow cytometry. Representative photographs of DAPI (blue) and TUNEL (pink) staining; scale bar = 25 μm. The data are expressed as mean ± SEM (*n* = 3). *p < 0.05 versus the NG group, ^#^*p* < 0.05 versus the HG group, ^&^*p* < 0.05 versus the HG group, and ^+^*p* < 0.05 versus the HG plus DATS group

A previous study has demonstrated that abnormal mitochondrial fission can cause the production of mitochondrial ROS [12]. We stained HUVECs with MitoTracker Red after different treatments and found that the mitochondria in the NG group mainly presented as elongated tubules, whereas the high-glucose culture conditions significantly altered the mitochondrial morphology in HUVECs. Notably, however, DATS treatment blocked the high glucose-induced mitochondrial fission and morphological changes (Fig. 4a, b).

To explore the mechanism by which DATS attenuates mitochondrial fission, we measured the level of Drp1 protein in HUVECs. Compared with the NG group, the HG group showed an increased expression of Drp1 protein, whereas DATS treatment suppressed high glucose-induced Drp1 expression in HUVECs, and the protective effect of DATS was abolished by overexpression of Drp1. However, knockdown of Drp1 expression using Drp1 siRNAs (Fig. 4e) could prevent high glucose-induced mitochondrial fission, downregulate the generation of cellular superoxide anions and mitochondrial ROS, and attenuate HUVEC apoptosis induced by high-glucose treatment (Fig. 4c–m).

### DATS blockage of high glucose-induced mitochondrial fission via AMPK activation

**Fig. 5:**
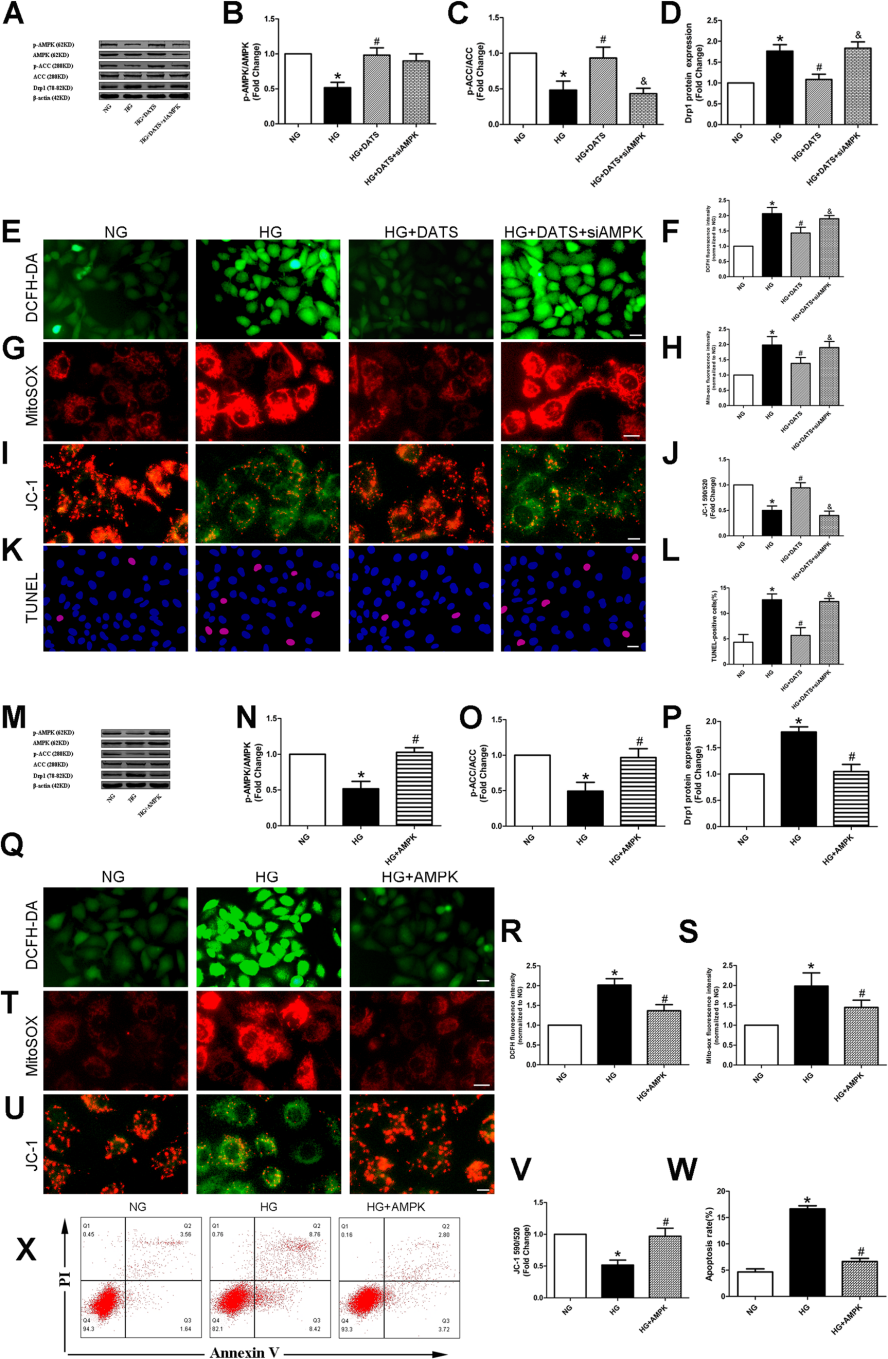
Effect of DATS on protection of high glucose-induced Drp1 and HUVEC mitochondrial fission via AMPK activation. **a**–**d**, **m**–**p** Western blot. HUVECs were seeded, treated with different agents, and subjected to western blot analysis of p-AMPK, AMPK, p-ACC, ACC, and Drp1 proteins. **e**, **g**, **q**, **t** Intracellular and mitochondrial ROS assay. **f**, **h**, **r**, **s** Quantified data described in (**e**, **g**, **q)**, and **t**. **i**, **u** JC-1 staining of the mitochondrial membrane potential. **j**, **v** Quantified data described in (**i**) and (**u**). **k**, **x** TUNEL assay and flow cytometry. **l**, **w** Quantified data described in (**k**) and (**x**). Representative photographs of DAPI (blue) and TUNEL (pink) staining; scale bar = 25 μm. The data are represented as mean ± SEM (*n* = 3). *p < 0.05 versus the NG group, ^#^*p* < 0.05 versus the HG group, and ^&^*p* < 0.05 versus the HG plus DATS group

AMPK has previously been illustrated to modulate Drp1 expression in many cell types [24, 25]. In addition, DATS is reported to protect against MI/R injury and cell apoptosis through AMPK activation [18]. Thus, in this study, we explored whether AMPK activation mediates the effects of DATS on the reduction in high glucose-induced mitochondrial fission by assessing the levels of phosphorylated AMPK and the downstream effector ACC. Our results showed that both proteins were downregulated in the HG group, compared with the NG group. In contrast, DATS treatment reversed the alterations in HUVECs cultured under high-glucose conditions, and the protective effect of DATS was abrogated by knockdown of AMPK (Fig. 5a–l). However, overexpression of AMPK in HUVECs upregulated the phosphorylation levels of AMPK and ACC, downregulated Drp1 protein expression, downregulated the generation of cellular superoxide anions and mitochondrial ROS, improved the mitochondrial membrane potential, and attenuated HUVEC apoptosis induced by high-glucose treatment (Fig. 5m–x).

## Discussion

Diabetes-induced blood vessel injury doubles the risk of cardiovascular disease [2] and is responsible for up to 75% of diabetic deaths [3]. Thus, the development of novel approaches will protect blood vessels from high glucose-induced endothelial cell damage. In our current study, we revealed that DATS was able to reduce high glucose-induced HUVEC apoptosis by blockage of high glucose-induced ROS generation in HUVECs. We also found that glucose-induced HUVEC apoptosis was via the mitochondria-related apoptosis pathway, whereas DATS could block this pathway efficiently in vitro. Moreover, DATS protected the mitochondria by maintenance of the mitochondrial membrane potential and inhibition of high glucose-induced Drp1 expression. Overall, the inhibition of high glucose-induced HUVEC mitochondrial apoptosis by DATS was via AMPK activation and, subsequently, suppression of Drp1 expression. Future studies will assess the effects of DATS in vivo.

It is well acknowledged that hyperglycemia is the single most important risk factor for the development of cardio-cerebrovascular complications in diabetic individuals [3]. Hyperglycemia can induce endothelial cells to undergo excessive apoptosis, which damages the integrity of the vascular endothelial layer and thereby triggers cardiovascular events, including atherosclerosis [26]. Previous studies have demonstrated that the treatment of endothelial cells with high glucose concentrations results in cell apoptosis [27–29]. Our current study confirmed this conclusion, and we also revealed that DATS was able to significantly reduce HUVEC apoptosis induced by high-glucose culture conditions, suggesting that DATS should be further evaluated as an effective antiatherosclerotic drug. Our current data are consistent with a recent study reporting that DATS exhibits an antiapoptotic effect in ischemic muscles in mice as well as in HUVECs treated with hypoxia and serum starvation to activate the Akt-eNOS signaling pathway [30]. However, another study has demonstrated that DATS can facilitate tumor cell apoptosis in vitro by activation of the MAPK signaling pathway [31], which is quite different from our current data and this previous study [30]. The reason for this discrepancy is unknown, but it may be due to the fact that DATS differentially regulates cell apoptosis in different tissues. Furthermore, oxidative stress is a major inducer of HUVEC apoptosis [32], and it has been shown that DATS treatment was able to mitigate doxorubicin-induced cardiomyocyte apoptosis by inhibiting ROS generation [33]. Our recent study demonstrated that DATS enhanced tissue angiogenesis in diabetic mice with tissue ischemia by modification of the oxidative stress levels [17]. Our current study confirmed the effects of DATS on attenuation of high glucose-induced HUVEC cytoplasmic and mitochondrial ROS production. However, our current data are not consistent with other studies showing that DATS can induce tumor cell apoptosis and ROS generation in human gastric carcinoma cells [34], glioma cells [35], and osteosarcoma cells [36]. These data speculate that the effects of DATS are cell type dependent, e.g., normal versus tumor cells, thus requiring further investigation. In addition, our current study revealed that Mito-TEMPO, a specific mitochondria-targeted antioxidant, showed an equal inhibitory effect on ROS generation in HUVECs as DATS, further suggesting that mitochondrial ROS was the main source of intracellular ROS to block high glucose-induced HUVEC mitochondrial apoptosis.

The recent literature reports indicate that mitochondrial injury and loss of the mitochondrial membrane potential are the early events of cell apoptosis, while mitochondrial injury depends on superabundant cellular ROS generation [37, 38]. Our current study demonstrated that DATS was able to block high glucose-induced loss of the HUVEC mitochondrial membrane potential and the expression of certain genes, such as cytochrome c and Bax, but Bcl-2 expression was upregulated in HUVECs, indicating that DATS could mitigate mitochondrial apoptosis. Indeed, the mitochondria are highly dynamic organelles and play a pivotal role in cell apoptosis [23, 39]; therefore, the suppression of mitochondrial fission was able to alleviate diabetes-accelerated endothelial cell apoptosis and atherosclerosis. In addition, accumulated evidence suggests that Drp1 triggers mitochondrial fission and apoptosis in mammalian cells [39–41]. The results of the current study are consistent with those of previous studies, i.e., the expression of Drp1 protein was upregulated in high glucose-cultured cells, whereas DATS treatment was able to block such an upregulation. Although previous studies have proposed non-Drp1-related mechanisms of mitochondrial fission [42, 43], the precise mechanism of Drp1 and DATS on mitochondrial fission needs further investigation.

AMPK is the key molecule that is primarily involved in regulating the mitochondrial energy metabolism [44]. A previous study has shown that AMPK activation is able to inhibit MI/R injury-induced cardiomyocyte apoptosis by suppression of mitochondrial fission [44]. Our current study confirmed this notion and showed that DATS activated AMPK-downregulated Drp1 expression and alleviated endothelial cell apoptosis under high-glucose conditions. However, the effects of DATS on mitochondrial activities might be multiple, well beyond its regulation of the mitochondrial dynamics. For example, Shimizu et al. [45] have demonstrated that AMPK activation by hydrogen sulfide enhanced the expression of PGC-1a, a regulator of mitochondrial biogenesis in cardiomyocytes. Therefore, the mitochondrial dynamics and mitochondrial biogenesis might both be potential contributors to the DATS-mediated mitochondrial adaptations. The current study was just a proof-of-principle study; thus, many more studies are needed to establish the role of DATS in preventing the diabetes-induced development of cardiovascular complications.

### Limitations

The current data were all obtained via a cell culture-based study. We only stimulated cells with HG to simulate hyperglycemia. It will be much more complicated in the human body. Moreover, a recent investigation has demonstrated that hyperglycemia is related to inflammation [46], but the related indicators of inflammation were not detected in our present investigation.

### Further investigations

The antiapoptotic mechanism by which DATS regulates various signaling pathways is still undetermined and needs to be clarified in the near future. In addition, further research is also needed to investigate the effects of DATS in vivo.

## Conclusion

In conclusion, our current study demonstrated that DATS was able to protect HUVECs from high glucose-induced apoptosis by blockage of high glucose-induced oxidative stress and mitochondrial fission through AMPK activation in HUVECs. A future study will assess the effects of DATS in vivo.
